# Neurocognitive follow‐up in adult siblings with Phelan–McDermid syndrome due to a novel *SHANK3* splicing site mutation

**DOI:** 10.1002/mgg3.1780

**Published:** 2021-08-09

**Authors:** Minna Kankuri‐Tammilehto, Oili Sauna‐aho, Maria Arvio

**Affiliations:** ^1^ Department of Clinical Genetics Turku University Hospital Turku Finland; ^2^ Institute of Biomedicine University of Turku Turku Finland; ^3^ KTO, Support and Expert Center for Persons with Intellectual Disability Southwest Special Care Municipal Authority Paimio Finland; ^4^ Neurology Päijät‐Häme Joint Municipal Authority Lahti Finland; ^5^ PEDEGO Oulu University Hospital Oulu Finland

**Keywords:** infantile autism, intellectual disability, mosaicism, Phelan–McDermid syndrome, *SHANK3* splicing site mutation

## Abstract

**Background:**

Phelan–McDermid syndrome (PMD) is usually not only caused by 22q13.3 deletion but also pathogenic variants (mutations) of *SHANK3* gene. PMD is characterized by global intellectual disability, severely delayed or absent speech, and features of autism spectrum disorder and susceptibility to psychotic behavior. Here, we describe a neurocognitive follow‐up and genetic etiology for two siblings with PMD.

**Method:**

Comparative genomic hybridization (CGH) array test was normal and no 22q13.3 deletion was observed. For this reason, whole exome sequencing (WES) analyzed the siblings’ and the parents’ DNA sample.

**Results:**

The results of the siblings strongly suggest that the *SHANK3* gene variant c.2313+1G>A is pathogenic and PMD can be inherited from a mosaic father for this gene variant. Both siblings learned new skills until puberty but experienced a neuropsychiatric disaster after the age of 14 years experienced neurocognitive decline and it was sharp for one of the siblings.

**Conclusion:**

The long‐term observations are sparse in PMD and *SHANK3* mutations. This is the neurocognitive follow‐up from childhood to middle ages, where a sharp neurocognitive decline was observed. We conclude that progressive neuropsychiatric symptoms in adolescence are a universal clinical clue for PMD diagnosis and an underlying *SHANK3* splicing site mutation.

## Graphical abstract

Here, we describe two siblings with a novel *SHANK3* gene variant of c.2313+1G>A which is inherited from the healthy mosaic father. The results of the siblings point that the variant is pathogenic. The long‐term observations are sparse in PMD and *SHANK3* mutations. This is the neurocognitive follow‐up from childhood to middle ages, where a sharp neurocognitive decline was observed.

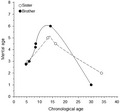

## 1 INTRODUCTION

People with an intellectual disability (ID) form a most heterogenic group of people regarding etiology, severity, and associated impairments of ID. Phelan–McDermid (PMD, OMIM 606232) or 22q13.3 syndrome was described in 1985 (Phelan et al., 2001; Sarasua et al., 2014; Watt et al., 1985) and according to the literature so far over 500 patients have been identified. In recent years, heterozygous *SHANK3* pathogenic variants (later referred as mutations) have been linked to autism spectrum disorder and ID (Boccuto et al., 2013). *SHANK3* mutations are inherited in an autosomal dominant manner; however, the majority of affected individuals represent de novo mutations (Phelan et al., 2018). It was quickly deduced that different mutations in *SHANK3* gene with autosomal dominant inheritance pattern can cause a similar phenotype to the 22q13.3 microdeletion (De Rubeis et al., 2018). A splicing site mutation is rare in PMD, 2% of all *SHANK3* mutations. We report two siblings with PMD due to a previously not a well‐known splice site variant in *SHANK3* gene and a more than 25 years neurocognitive follow‐up.

## 2 METHOD

### 2.1 Ethical compliance

The study complies with the Declaration of Helsinki regarding the use of human samples and identifiable information. This study is a case report and informed consent was obtained from the patients regarding the use of information obtained during clinical treatment.

### 2.2 Family description

**FIGURE 1:**
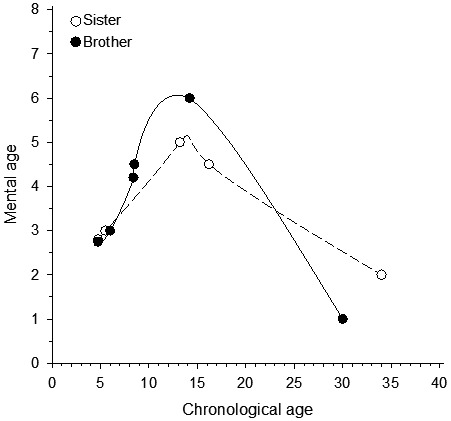
Mental ages of the patients according to chronological age

The parents are academically educated. The 68‐year‐old father is otherwise healthy but has undergone cancer treatments. The 65‐year‐old mother is healthy and there is no consanguinity. There are three children in the family. The eldest one is healthy and two others are PMD patients: currently a 37‐year‐old daughter and a 30‐year‐old son. Both patients have had normal pre‐ and perinatal history. They learned to walk at the age of 16 months. Because of delayed development, they both were referred to a child neurological consultation at the age of 4 each. Clinical medical examination showed neither dysmorphic features nor physical abnormalities. Etiological studies (standard karyotype, fragile X DNA, and metabolic screening) did not reveal any cause of delayed development. Since childhood they have been assessed five times with neuropsychological and linguistic test methods (Figure 1).

### 2.3 The sister

The sister, at the age of 4, was described as strongly attached to her parents, and having echolalia. Psychological test according to Merrill–Palmer (Gordon, 1933) referred to mild ID. At the age of 5, she spoke short sentences, test results indicated moderate ID according to Merrill–Palmer (Gordon, 1933; Kuusinen & Blåfield, 1974), Bo Ege (Ege, 1985), and Van Alstyne (Van Alstyne, 1961). At the age of 13, she was described as joyful and being well in contact and concentrating in psychological tests WISC‐R (Wechsler, 1984), NEPSY (Korkman, 1988, 1997), and ITPA (Stewart, 1977; Sutter & Bishop, 1986), which revealed moderate ID nonverbal reasoning being stronger than verbal. She could not orientate to time or place. She had learned to speak and dress herself but needed help in toileting and in taking care of personal hygiene. At the age of 16, her cognitive skills according to WPPSI‐R (Wechsler, 1989), Merrill–Palmer (Gordon, 1933), and MVPT (Colarusso & Hammill, 1972) were at the same level as at the age of 13. She had severe problems in executive functions and generalization of learned skills. Between ages 16–33 years, there is no medical or psychological data recorded. Growth parameters such as height, weight, and head circumference, were normal.

At the age of 33, she had single generalized tonic–clonic seizures and valproate was initiated but terminated soon because of psychic adverse effects. Epileptic seizures re‐appeared at the age of 36 as generalized tonic–clonic seizures followed by postictal hemiparesis on right side as well as prolonged absences. EEG recording showed slow wave focus on left side. Brain CT finding was normal. Levetiracetam, lacosamide, and zonisamide gave no response and were withdrawn because of severe adverse effects; psychotic and manic‐depressive behavior, insomnia, and bruises in skin which appeared and diminished rapidly without any clear cause. After initiation of clonazepam 1 mg/day, the seizures ended as well as psychotic behavior. As a tranquillizer, she has benefitted from a small dose of oxazepam (7.5 mg/day). When falling to sleep she tends to cough at lot and secrete mucus. She has distal biting and mild swayback. At the age of 37, we organized a neuropsychological assessment. She spoke and asked questions like “who are you.” Unfortunately, testing failed because she could not concentrate on required tasks. According to interviews and clinical observations her skills correspond to the level of severe/profound intellectual disability.

### 2.4 The brother

The brother, at the age of 4, spoke short sentences and answered to simple questions. Neuropsychological and linguistical tests according to Leiter (Leiter, 1979), Merrill–Palmer (Gordon, 1933; Kuusinen & Blåfield, 1974), VMI (Beery, 1989), and Reynell (Reynell & Huntley, 1987) referred to mild ID. The developmental profile was quite even. At the age of 6, the Reynell linguistical test (Reynell & Huntley, 1987) showed slight development of verbal skills. At the age of 8, he was described as restless and having poor concentration skills and results of psychological assessment according to WPPSI‐R (Wechsler, 1989), Nepsy (Korkman, 1988, 1997), and ITPA (Stewart, 1977) indicated moderate ID. At the age of 14, he managed to concentrate on the required tasks. His verbal reasoning was stronger than non‐verbal, and his verbal skills corresponded the level of moderate ID, whereas nonverbal skills corresponded severe ID according to WPPSI‐R (Wechsler, 1989) and Nepsy (Korkman, 1988, 1997). He could read and write and mastered simple sums.

At the age of 15 years, he became psychotic and was referred to hospital care. He was treated with risperidone, haloperidol, and benzodiazepines. Two months later, he started to have single generalized tonic–clonic and absence seizures. EEG showed temporal slow wave focus on right side and brain CT was normal. No antiepileptic treatment was started.

At the age of 16, he underwent ADOS and ADI‐R assessments which revealed autism spectrum disorder manifesting as repeated stereotypic behaviors, disturbances on mutual communication, and poor communication skills. Psychological assessment failed because of poor co‐operation. He was diagnosed having unspecified mood disorder with manic symptoms. Growth parameters such as height, weight, and head circumference, were normal.

At the age of 25, he was very thin and very restless, slept only a few hours a day, made noises, jumped, and stopped when parents fed him. Brain MRI was normal and array comparative genomic hybridization (CGH) did not detect copy number variations. Celiac disease was excluded, but since his condition impaired, casein‐ and gluten‐free diet were experimentally initiated. Five years later it was estimated that the patient had benefited from the diet and was less anxious than before the diet. Still, he kept on moving all the wake time, clapped and rubbed hands, slept on average 4 hours a day, did not speak, needed constant guidance but was able to bike together with an accompanying person in rural streets. As his sister, he is on oxazepam (7.5 mg/day). He has no dysmorphic features, but mild scoliosis. Since the age of 15, no seizures have been observed. Neuropsychological test ITPA (Stewart, 1977) and WPPSI‐R (Sutter & Bishop, 1986; Wechsler, 1989) revealed profound ID.

### 2.5 Genetics

CGH array test was normal for both siblings and no 22q13.3 deletion was observed. For this reason, whole exome sequencing (WES) analyzed the sisters’ and the parents’ DNA sample.

## 3 RESULTS

### 3.1 Result of neurocognitive follow‐up

Figure 1 shows the neurocognitive development of both siblings compared to reference range at different ages. Both siblings experienced neurocognitive decline and it was sharper for the brother.

### 3.2 Result of genetic studies

CGH array test was normal and no 22q13.3 deletion was observed. Whole exome sequencing (WES) analyzed from the siblings’ and the parents’ DNA sample, showed in the *SHANK3 gene* (SH3 and multiple ankyrin repeat domains 3) a heterozygous splicing site mutation c.2313+1G>A, in intron 20 (GenBank reference sequence NM_001080420.1). The variant was confirmed by Sanger sequencing. The substitution is located in the donor splice site of intron 20. Alamut Genova, an advanced variant exploration, defines that the consequence of this change is not predictable, but a skip of exon 20 is highly likely. The variant is predicted to disrupt the highly conserved donor splice site of exon 20. The frequency of the variant in the population is extremely low (0.000012 gnomAD) also in Finland as the variant is not observed in SISU (The Sequencing Initiative Suomi) database. ClinVar definition from Baylor Genetics as a single submitter interprets this variant as pathogenic. An updated classification (May 2020) of this variant due to guidelines proposed by the American College of Medical Genetics and Genomics (ACMG) (Richards et al., 2015) is Class 3––Unknown pathogenicity. The same variant has been found in the healthy father who carries a genetic mosaicism with approximately 13% mutated cells in blood lymphocytes.

## 4 DISCUSSION

We organized next‐generation sequencing studies for the siblings due to the history of the ID with rapid loss of cognitive functions from the puberty onward accompanied with focal onset secondarily generalized epileptic seizures and psychosis. Whole exome sequencing (WES) found *SHANK3* gene mutation named c.2313+1G>A from the siblings’ DNA sample, and from healthy father as mosaic (13% of blood lymphocytic cells). Previously, in literature, *SHANK3* pathogenic variant has been shown to inherit from the mother (Verhoeven et al.,^2020^). The results of this family bring up that the inheritance of *SHANK3* pathogenic variant from a mosaic father is possible in PMD. The father is healthy and has had no symptoms of PMD.

So far, more than 210 different *SHANK3* mutations have been found, but so far only 4 are splicing site mutations. The amount of observed splicing site mutation types is 2% of all *SHANK3* mutations types. Our results confirm that the variant c.2313+1G>A, in intron 20 is the fifth known splicing site mutation in *SHANK3 gene*.

The sibling's symptoms match well with PMD. Before preschool age, they showed delayed development manifesting in social, communication, self‐help, and cognitive skills. They learned new skills until puberty, but both experienced a neuropsychiatric disaster after the age of 14 years. The brother's crisis began with psychosis followed by epileptic seizures. The sister's documented progression at the age of 33 started with epileptic seizures followed by manic psychosis. Traditional antiepileptic drugs and neuroleptics worsened their condition. However, both have benefited from small doses of benzodiazepines and the brother also from casein‐ and gluten‐free diet.

These are symptoms that have previously often been seen in PMD (De Rubeis, 2018; Kohlenberg et al., 2020; Verhoeven et al., 2020). Pathogenic variants of the *SHANK3* gene can cause a similar phenotype as the 22q13.3 microdeletion (De Rubeis et al., 2018). The variant is still not classified as pathogenic variant according to the ACMG guidelines (Holder & Quach, 2016; Richards et al., 2015). The findings in our siblings strongly suggest that the observed *SHANK3* variant is a significant pathogenic variant, and the mutation that caused our patients’ symptoms. The long‐term neuropsychiatric observations are sparse in PMD and SHANK3 mutations although there are some reports of adulthood PMD due to 22q13.3 deletion and SHANK3 mutation (Verhoeven et al., 2013, 2020).

To the best of our knowledge, this is the first follow‐up genotype–phenotype correlation description in patients with c.2313+1G>A *SHANK3* mutation. We have been able to follow the clinical course and neurocognitive decline of two siblings from childhood to middle ages. One sibling's decline has been sharp.

We conclude that progressive neuropsychiatric symptoms in adolescence are a universal clinical clue for PMD diagnosis and an underlying *SHANK3* splicing site mutation.

## CONFLICT OF INTEREST

The authors declare no conflict of interest.

## AUTHOR CONTRIBUTIONS

Minna Kankuri‐Tammilehto: Performed clinical genetic analysis and participated in writing the paper. Maria Arvio: Participated in neurocognitive analysis and participated in writing the paper. Oili Sauna‐aho: Participated in neurocognitive analysis and participated in writing the paper.

## ETHICS STATEMENT

The study complies with the Declaration of Helsinki regarding the use of human samples and identifiable information. This study is a case report and informed consent was obtained from the patients regarding the use of information obtained during clinical treatment. In the study analyzed data was from patients who had been treated at the hospital. As no new samples were required a separated ethics board permit was not required.

## Data Availability

The data that support the findings of this study are available from the corresponding author upon reasonable request.
